# The MultiOmics Explainer: explaining omics results in the context of a pathway/genome database

**DOI:** 10.1186/s12859-019-2971-6

**Published:** 2019-07-18

**Authors:** Suzanne Paley, Peter D. Karp

**Affiliations:** Bioinformatics Research Group, SRI International, 333 Ravenswood Ave, Menlo Park, 94025 CA USA

**Keywords:** Network analysis, Pathfinding, Pathway visualization, Multiomics analysis, Pathway/genome database

## Abstract

**Background:**

High-throughput experiments can bring to light associations between genes, proteins and/or metabolites, many of which will be explainable by existing knowledge. Our aim is to speed elucidation of such explanations and, in some cases, find explanations that scientists might otherwise overlook.

**Results:**

We describe the MultiOmics Explainer, a new tool within the Pathway Tools software suite that leverages what is known about an organism’s metabolic and regulatory network to suggest explanations for the results of omics experiments. Querying a database such as EcoCyc, the MultiOmics Explainer searches the organism’s network of metabolic reactions, transporters, cofactors, enzyme substrate-level activation and inhibition relationships, and transcriptional and translational regulation relationships to identify paths of influence among input genes, proteins and metabolites. Results are presented in a combined metabolic and regulatory diagram. We present several examples of explanations generated for associations found in the *Escherichia coli* literature.

**Conclusions:**

The MultiOmics Explainer is a valuable tool that helps researchers understand and interpret the results of their omics experiments in the context of what is known about an organism’s metabolic and regulatory network. It showcases the rich set of computational inferences that can be drawn from a database such as EcoCyc that encodes a diverse range of biological interactions.

**Electronic supplementary material:**

The online version of this article (10.1186/s12859-019-2971-6) contains supplementary material, which is available to authorized users.

## Background

High-throughput experiments can bring to light associations between genes, proteins and/or metabolites whose relationship is not immediately obvious to researchers. Some of these associations reveal new functions or pathways; but in a well-studied organism, such as *Escherichia coli*, many of these associations will be explainable by existing knowledge. However, finding such explanations can be time consuming for scientists, because, in some cases, long chains of interactions connect causes with their effects. Our aim is to speed elucidation of such explanations, and, in some cases, find explanations that scientists would otherwise overlook, to aid researchers in differentiating which effects can and cannot be explained by existing knowledge.

Here we describe the MultiOmics Explainer, a new tool within the Pathway Tools software suite that leverages what is known about an organism’s metabolic and regulatory network to suggest explanations for the results of omics experiments. The MultiOmics Explainer is unique in that it synthesizes the wide range of knowledge contained within a Pathway/Genome Database (PGDB), including the organism’s metabolic network, transporters, cofactors, enzyme substrate-level activation and inhibition, and transcriptional and translational regulation. The MultiOmics Explainer is also unique in that its inputs can come from multiple types of omics experiments, including transcriptomics, proteomics, metabolomics, and combinations thereof. It should be noted that this tool does not attempt to infer any previously unknown regulatory or other relationships—it uses strictly what is already known and encoded in the PGDB. Thus, the failure of the tool to find a plausible explanatory relationship in any particular case is a likely indication of gaps in the metabolic or regulatory network, and suggests areas for new research or curation.

**Fig. 1:**
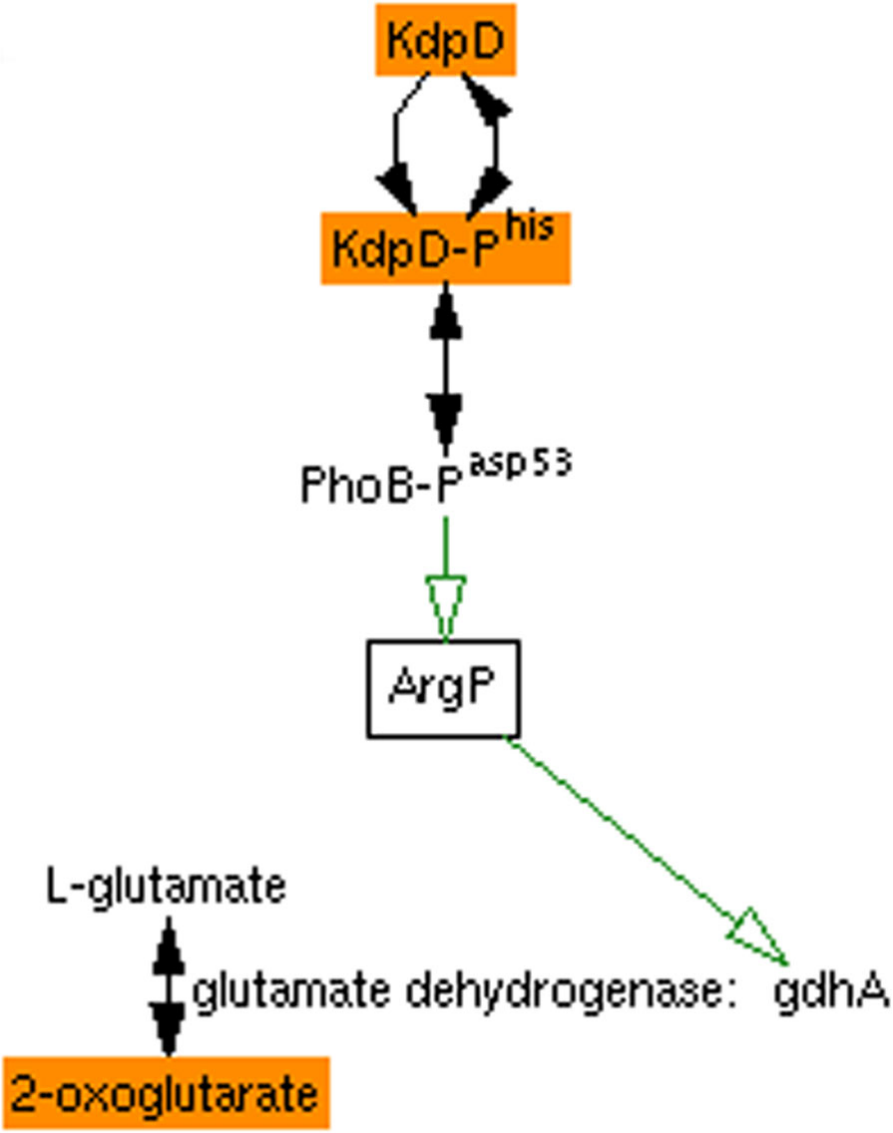
A diagram generated by the MultiOmics Explainer suggesting a path by which *E. coli* KdpD affects 2-oxoglutarate (both shown in orange). Green arrows represent activation, and black arrows represent chemical reactions

For example, suppose an omics experiment found that knocking out the *E. coli* sensor histidine kinase gene kdpD resulted in an increase in the level of 2-oxoglutarate in the cell (such a relationship has in fact been found [1]), but it was not immediately obvious to the researcher how they might be related. The MultiOmics Explainer suggests a likely mechanism based on the interactions found in EcoCyc (phosphorylated KdpD transfers its phosphate group to PhoB, which activates expression of transcriptional regulator ArgP, which activates expression of glutamate dehydrogenase gdhA, which catalyzes a reaction that interconverts glutamate and 2-oxoglutarate), and presents it as the easy-to-understand diagram shown in Fig. 1.

Pathway Tools [2] is a software environment for generating, maintaining, analyzing, visualizing, and web-publishing Pathway/Genome Databases. A PGDB is a model organism database that combines an organism’s genomic information with what is known or inferred about the organism’s metabolic and transport network, including chemical reactions, metabolites and pathways; and what is known about the organism’s regulatory network, including operons, transcription factors, translational regulators, and substrate-level enzyme modulation. EcoCyc [3] is a PGDB for *E. coli* K–12 that has been curated extensively from the biological literature, integrating information from more than 37,000 publications. Because EcoCyc is the most complete and most accurate PGDB in the BioCyc collection [4], with the most complete set of regulatory interactions of all kinds, it serves as the optimal database on which to test the MultiOmics Explainer. Although the tool can be run on any PGDB within the BioCyc collection, to the extent that the regulatory network in other PGDBs is less complete or absent, results may be less useful. Additional file 1: Table S1 lists the organisms containing the largest regulatory networks within BioCyc. All of these regulatory networks were captured through literature-based curation by BioCyc, or imported from DBTBS [5] or RegTransBase [6].

The MultiOmics Explainer can operate in one of two modes. In **directed mode**, the user specifies two sets of target entities, the **conditions** and **effects**, where an entity is any gene, protein, or metabolite of interest. The conditions are a set of one or more entities whose abundance or activity has been changed as part of the conditions of the experiment (e.g. knocked-out genes, or metabolites supplied as nutrients). The effects are a set of one or more entities whose abundance or activity has significantly changed as a result of the experiment. For every possible condition-effect pair, the tool will attempt to find the lowest cost paths through the combined metabolic and regulatory network that link the condition to the effect.

In **undirected mode**, the user specifies a single set of target entities, the **effects** set. The tool will attempt to find paths that link the effects together by identifying one or a small number of entities, **influencers**, that influence multiple targets. These influencers may or may not themselves be members of the effects set.

We treat both modes as network-search problems, using a weighted breadth-first search approach with a maximum cutoff to explore the combined metabolic and regulatory space. The weighting of a connection depends both on the connection type (e.g., we use a different formula for enzymes and substrates of producing or consuming reactions vs. regulators of various kinds) and specificity (e.g., an edge to a metabolite that participates in a large number of reactions or to a transcription factor that regulates a large number of genes will be assigned a higher cost than an edge to a metabolite that participates in a small number of reactions or a more specific transcription factor).

An additional challenge is to present the results of this analysis in a form that is easily comprehensible to biologists. This task is complicated by the diversity of relationship types that need to be communicated to the user. Our tool presents its results as a combined metabolic and regulatory network diagram, with mouseovers that more fully describe the various relationships. If, due to a large number of interconnections, this generated diagram becomes difficult to understand, the user can interactively select subsets of the original entities to examine different subsets of relationships, a few at a time.

Finally, we present some examples of the use of the MultiOmics Explainer to explain the results obtained from previously published *E. coli* omics studies.

### Related work

Most prior work focuses on analyzing either gene/protein data and networks or metabolite data and networks, but not both together.

Typical analysis of transcriptomics data involves statistical methods, such as enrichment analysis, sometimes combined with topological pathway data, to identify pathways or Gene Ontology terms that show significant differential expression (reviewed in [7]). CliPPER [8] uses Gaussian graphical models to select pathways with significant differential expression and then to identify the portions of those pathways most correlated with phenotype. Sub-SPIA [9] combines impact analysis [10] with minimum spanning trees to identify significantly perturbed sub-pathways. Note that the pathways used in these analyses are signaling pathways, not metabolic pathways. ScorePAGE [11] uses metabolic pathway topology to weight enzyme pairs in score calculations by their metabolic distance. These approaches are significantly different from ours in that they are not primarily based around analysis of metabolic or regulatory networks. They use pathway data primarily as a means of grouping genes into functionally related bins, as opposed to our approach which is based on graph traversal. Even those approaches that incorporate network topology only consider it within the context of predefined pathways that have already been identified as differentially expressed. Thus, they identify already known pathways whose expression changes, and then use the network to narrow the explanation down to portions of those pathways, rather than identifying causal explanations from within a large network. They also offer no means to explicitly link results to experimental conditions, as in our directed mode.

On the metabolomics side, Acuna et al. [12] have developed techniques to derive a set of maximal directed acyclic subgraphs of a metabolic network, which constitute a set of metabolic “stories” explaining how the metabolites are interconnected. These stories can then be prioritized on the basis of metabolomics data, and multiple related stories can be collected into an “anthology” [13]. This work is somewhat similar to ours in that it considers the organism’s entire metabolic network and generates fully instantiated paths linking entities of interest. Our approaches to graph construction are quite different however—their approach is more computationally demanding but results in a complete set of possible paths (which then must be prioritized), whereas we use cutoffs and other heuristics to reduce computation time and limit the number of paths produced.

Our work differs from all the above techniques in that it considers multiomics data and draws on a much richer set of biological relationships, combining the metabolic network with multiple different types of regulation.

## Implementation

**Fig. 2:**
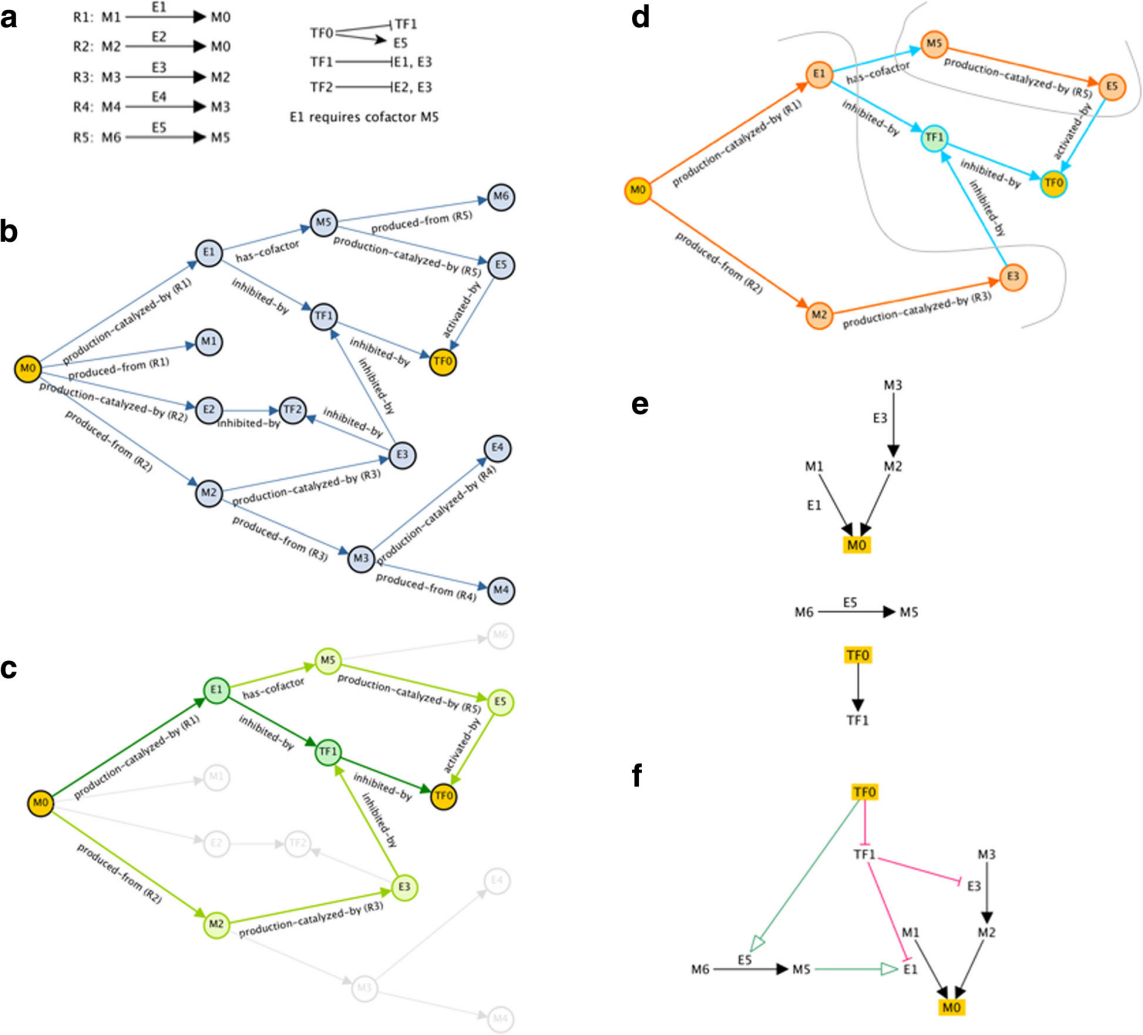
A simplified example showing the three phases of the algorithm. In this example, the user has specified a single condition, transcription factor TF0, and a single effect, metabolite M0, both colored yellow throughout. **a** A simple metabolic and regulatory network consisting of five enzyme-catalyzed reactions and three transcription factors. E1 catalyzes the conversion of metabolite M0 to metabolite M1. Transcription factor TF0 inhibits expression of transcription factor TF1, and activates expression of enzyme E5. **b** The graph *G*1 generated during Phase 1. For this simplified example, assume all edge weights are 1. In actual operation, the edge weights would be determined by the formulae in Table 1. **c** In Phase 2, we search for paths connecting M0 and TF0. The shortest path is shown in dark green, but the paths shown in light green are only slightly longer, so all three are kept. The filtered graph *G*2 contains only the colored nodes and edges. **d** In Phase 3, considering only the edges associated with reactions (colored orange), we divide the graph into connected components, and create two temporary pathways, one consisting of reactions *R*1,*R*2 and *R*3, and the other consisting of just *R*5. The remainder of the graph (colored blue) is also divided into connected components. **e** Each of the components is laid out individually. We use Pathway Tools’ automated pathway layout algorithms for the temporary pathways, suppressing display of any enzymes or side metabolites that are not part of *G*2. **f** The individual components are reassembled and appropriate node and edge styles assigned to form the Explanation Graph *G*3

The MultiOmics Explainer algorithm is divided into three phases, which can be roughly categorized as graph construction, graph search, and network visualization (although phases 2 and 3 also involve generation of working graphs). Figure 2 illustrates each of these phases using a simplified example network.

**Table 1:** The types of influences *I* on an entity *X* and their edge costs

TYPE OF INFLUENCER	EDGE COST FORMULA
**X is a reaction substrate** (including proteins in ligand-binding reactions) ^a^:	
Reactant *I* of reaction producing *X*	1+(|*R*_*X*=*sub*_|+|*R*_*I*=*sub*_|)/20
Reactant *I* of reaction consuming *X*	1+(|*R*_*X*=*sub*_|+|*R*_*I*=*sub*_|)/10
Product *I* of reaction consuming *X*	2+(|*R*_*X*=*sub*_|+|*R*_*I*=*sub*_|)/10
Enzyme *I* of reaction producing *X*	1+|*R*_*I*=*enz*_|/20
Enzyme *I* of reaction consuming *X*	2+|*R*_*I*=*enz*_|/20
Transporter *I* of *X*	1+|*R*_*I*=*transporter*_|/20
**X is a gene, gene product, or protein complex**:	
Activator or inhibitor *I* of enzyme *X*	1+|*Enzs*_*I*=*modulator*_|/20
Cofactor *I* of enzyme *X*	1+|*Enzs*_*I*=*cofactor*_|/20
Transcriptional regulator *I* of *X*	1+(|*Regs*_*X*_|+|*G*_*I*=*reg*_|)/20
Translational regulator *I* of *X*	1+(|*Regs*_*X*_|+|*G*_*I*=*reg*_|)/20
Sigma factor *I* for transcription of *X*	1+|*G*_*I*=*reg*_|/20
Component *I* of protein complex *X*	0.1
Transcription unit *I* of gene *X*^b^	0

|*R*_*X*=*sub*_| should be read as the number of other reactions in which *X* is a substrate (reactant or product), and so forth. Other abbreviations: G=genes, Enz=enzyme, Reg=regulator.

^a^We use the term substrate to refer to both reactants and products of a reaction. We exclude certain compounds that appear in very large numbers of reactions, such as water, ATP, etc. Also, if *X* is a small molecule, we exclude reactions in which proteins bind *X*, on the grounds that such interactions typically affect the quantity or activity of the protein but not, to any appreciable extent, the small molecule.

^b^While transcription units are not among the classes of entities we handle, are not added to the queue, and are therefore dead-ends in the graph, these are useful interactions to add in undirected mode because they explain why two genes might be correlated.

### Algorithm inputs and outputs

**Fig. 3:**
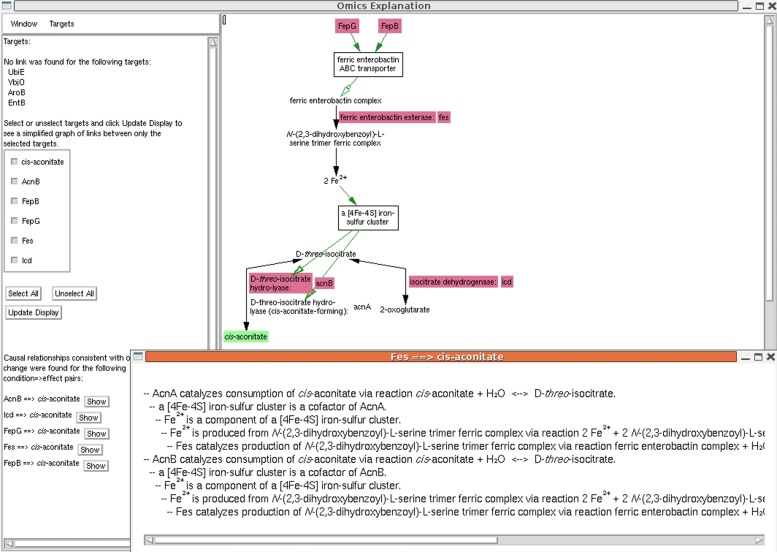
An example of the MultiOmics Explainer operating in directed mode on data from [1], showing genes whose knockouts led to measurement of increased levels of metabolite *cis*-aconitate. The control panel to the left of the display enables users to select a subset of entities for display, and indicates condition-effect pairs for which one or more consistent paths were found. Consistent paths connecting protein Fes to *cis*-aconitate are described in the popup window

The input to the MultiOmics Explainer is either one or two target sets of genes, proteins, and/or metabolites of interest. In undirected mode, a single set of entities is specified—these are the effects observed in the experiment. In directed mode, the user must specify both a set of condition entities and a set of effect entities. The total number of supplied entities should be kept fairly small, both for the analysis to complete in a reasonable amount of time and for the resulting diagram to be comprehensible. The target entities could be those exhibiting the largest or most significant change over the course of an experiment, or they could be hand-selected by a researcher who wants to understand their particular relationships. Note that it is possible to run the Phase 1 analysis on a larger set of entities, and then interactively select smaller subsets for Phases 2 and 3 to view in the diagram, particularly if the effect entities are genes (gene regulatory networks tend to be substantially sparser than metabolite networks) or little-used metabolites. The largest input set on which we have tested the MultiOmics Explainer consisted of 88 *E. coli* genes as effect entities and 1 condition entity, which required approximately 7 minutes to complete on a Linux desktop machine. Of the examples described in the “Results” section, the directed mode example shown in Fig. 3 with 9 condition entities and a single E. coli metabolite as the effect entity took only 11 seconds, and the undirected mode example with 25 E. coli genes completed in only 51 seconds. However, if the effect entities include metabolites that appear in large numbers of reactions, the analysis can take much longer. Analysis of an input dataset consisting of 55 common *E. coli* metabolites as effect entities and 1 gene condition entity required 45 min to complete.

For each entity (both conditions and effects), the user can optionally specify whether its abundance or activity is increased or decreased. If provided, this information will be used to color the resulting display, and to determine whether a given path is consistent with the observations or not (for example, if *A* increases and *B* decreases and *A* directly activates *B*, then the increase in *A* does not explain the decrease in *B*). Information about direction of change of an entity may also be used during Phase 2, to reduce the number of included paths.

The output of the MultiOmics Explainer is a diagram such as shown in Fig. 1d that illustrates one or more possible routes by which the condition entities (in directed mode) or identified common influencer entities (in undirected mode) influence the effect entities. The diagram is interactive, providing additional information on mouseovers, and links to the relevant entities in the Pathway Tools Navigator.

### Phase 1: generating the complete MetReg graph

The first phase of the algorithm is to build a weighted MetReg graph *G*1 of all influences on the set of effect entities, out to a specified maximum depth or cost *C*_*max*_. The maximum cost is a parameter to the algorithm. It does not correspond to any precise physical meaning but can be considered an approximate upper limit on the number of steps in the network connecting condition and effect. This parameter defaults to 20. Nodes in *G*1 are entities (e.g. proteins, metabolites), and edges correspond to influence types (e.g. produced-from, activated-by) that are computed from information stored in the PGDB. Note, however, that no direct one-to-one mapping exists between the objects and attributes in the PGDB and the nodes and edges in the MetReg graph. Although they are represented using different objects in the PGDB, for the purposes of this algorithm we consider a gene, its product, and any homomultimeric complexes of the product to be a single logical entity, so they are represented by a single node (chemically modified forms of an entity, such as a phosphorylated protein, are considered distinct objects, however, so different nodes are created for them). A single edge in the MetReg graph can combine information across multiple PGDB objects and attributes. For example, we create a single edge linking a metabolite to the enzyme that catalyzes its production, but to generate that edge we must draw information from attributes connecting the metabolite to a reaction object, the reaction to its catalysis object, and the catalysis object to the protein, as well as the directional information stored for the reaction.

The types of influences on an entity that we consider, and which map to edge types in the MetReg graph, are presented in Table 1. If the influencer is one of the input entities (condition or effect), then the edge weight to that entity will be 0.1. This will bias the algorithm to favor connections to the entities of interest. Otherwise, we use the formula listed in the table for that influence type. Most edge weights are 1 plus an additional factor that depends on the type and specificity of influence. This weighting is necessary to keep the size of the explored space tractable, but also maps to the biological intuition that action through global regulators and very common metabolites are less likely to explain the relationship between two entities if a more specific relationship can be found. Croes et al. [14] found that weighting metabolites by the number of reactions they participate in and using a lightest-weight-path algorithm led to more biologically significant routes through metabolic networks than naive shortest-path algorithms. We hypothesize that similar reasoning will apply with respect to MetReg networks. We also bias the weights to favor upstream effects (i.e., those that influence production of a metabolite, over those that influence consumption), because doing so produced more plausible results. The danger of this specificity-based approach is that it can miss biologically relevant paths of influence that involve common core metabolites that participate in many reactions such as pyruvate, or global regulators, such as Crp, but this trades off with the danger of having results be dominated by such paths even when they are not biologically relevant. The formulae for edge weights are designed to be fast to compute and have not yet been highly tuned. It is probable that these formulae could be further optimized.

We build the MetReg graph in breadth-first order, maintaining a priority queue *Q* to choose the next node to process. Each node in the graph keeps track of its lowest-cost path from each root node.

The graph-generation algorithm is as follows. Its inputs are the effect entities and the PGDB. Its output is a graph whose nodes correspond to database objects such as genes, metabolites, protein complexes, etc., and whose edges are influence relationships with associated costs stored at each node. Each node *N* has a cost *C*_*N*_, the cost of the minimum-weight path from *N* to any root, and one or more path costs *PC*_*N,R*_, the cost of the minimum-weight path from *N* to root node *R*. Each edge *N*→*N*^′^ has an edge cost $\phantom {\dot {i}\!}C_{N \rightarrow N'}$ as determined by the formulae in Table 1.

1. For each effect entity, create a corresponding root node *R* in *G*1 with path cost *PC*_*R,R*_=0, and add it to *Q* with cost *C*_*R*_=0.
2. Remove the lowest-cost node *N* from *Q*.
3. Query the PGDB to determine the set of metabolic and regulatory influencers *N*^′^ of *N* (see Table 1).
4. For each *N*^′^:
  Compute edge cost $\phantom {\dot {i}\!}C_{N \rightarrow N'}$.
  If node *N*^′^ does not yet exist in *G*1 and $\phantom {\dot {i}\!}C_{N}+C_{N \rightarrow N'}<C_{max}$, create node *N*^′^ and add edge *N*→*N*^′^. Set $\phantom {\dot {i}\!}C_{N'}=C_{N}+C_{N \rightarrow N'}$. Add *N*^′^ to *Q*.
  If node *N*^′^ already exists in *G*1, add edge *N*→*N*^′^. Set $\phantom {\dot {i}\!}C_{N'}=min(C_{N'}, C_{N}+C_{N \rightarrow N'})$.
  For every root *R* that *N* has a path to, set $\phantom {\dot {i}\!}PC_{R,N'}=PC_{R,N'}+C_{N \rightarrow N'}$, unless *N*^′^ already has a lower-cost path to *R*. If any path lengths were added or updated, recursively propagate the updates to all child nodes of *N*^′^.
5. While there are nodes remaining on *Q*, go to step 2.

For this phase of MetReg graph construction, except insofar as they affect edge-weighting, the condition entities are not treated any differently than other potential influencers. Figure 2b shows an example graph generated during this phase from a very simple metabolic and regulatory network. The cost of building the MetReg graph depends primarily on the number of effect entities, and the size and connectivity of the regulatory and metabolic network in the vicinity of the effect entities.

### Phase 2: generating a filtered graph

In the second phase, we create a new filtered graph *G*2 that contains a subset of the nodes and edges in *G*1. In directed mode this graph only includes paths from effect entities to condition entities. For every condition entity, if a node for that entity exists in *G*1, then a path was found to it from one or more effect entities, and the minimum path length will be stored with the node. For each such effect/condition pair, we traverse *G*1 to find all paths between them whose cost is less than the minimum path length plus some offset. An offset is added because multiple ways could exist in which one entity influences another, and since our weighting algorithm is blunt and imprecise, there is no guarantee that the absolute lowest-weight path is the most biologically significant. If this results in too many paths, we can reduce the offset and/or filter the paths to only include those whose parity is consistent with the specified directions of change of the entities (if such information was provided). All nodes and edges on the selected paths are copied to *G*2. Figure 2c illustrates the filtering of the complete graph to the nodes and edges that comprise the shortest path between condition and effect, plus two additional paths that are just one step longer. The cost of filtering the graph will be proportional to the product of the number of effect entities, the number of condition entities, and the size and connectivity of the MetReg graph.

In undirected mode, in addition to looking for causal connections between one entity and another, we are also interested in finding PGDB entities not in the original target set that can influence multiple target entities, and so provide an explanation for why they are correlated. For each node *N* in *G*1 that has paths to two or more target entities, we compute its influence score as the sum of the minimum path length to each target, plus a penalty of 1.5 times the maximum depth limit for every target that *N* is not connected to. The penalty ensures that, all else being equal, entities that influence a greater number of target entities will have lower influence scores (where a lower score means greater influence) than entities that influence fewer target entities. We then sort the nodes in order of increasing influence score, and generate a simple covering set of influencers, via the following algorithm: examine the influencer nodes in order of increasing score. If an influencer node adds one or more targets to the list of covered targets, add the influencer node to the covering set, and its list of influenced targets to the list of covered targets. Continue until all targets are included in the covered target set, or until no influencer nodes remain. Note that this is not a minimal covering set—we consider it more useful to include the better-scoring influencers, even if that introduces some redundant coverage. The covering set may or may not include members of the target set.

Once we have determined the covering set of influencers, we have reduced the undirected-mode problem to the directed-mode problem, positing the covering set influencers as conditions, and can generate the filtered graph *G*2 as for directed mode.

### Phase 3: displaying the results

Once the filtered MetReg graph has been generated, we use it to generate a single diagram that combines and explains all mechanistic influences found by the algorithm. The diagram should be easy to read and intuitive for biologists to follow, which is a challenge, given the mixture of different types of interactions (if generating a single easy-to-read diagram combining all conditions, effects, and the paths between them is impossible, we also provide the option to regenerate the diagram for a subset of condition-effect pairs, as described in the next section). There is not necessarily a one-to-one correspondence between the set of nodes in the filtered graph and the set of entities visible in the diagram. For example, a particular path of influence may include the impact of an enzyme on a reaction product but not the reactants of the reaction, whereas in order for the figure to be comprehensible to biologists, we will want to show the reaction as a link from reactants to products, and then show the enzyme associated with that reaction, drawn as if it were part of a typical metabolic pathway.

Our approach is to lay out the diagram hierarchically. We divide the nodes in the filtered graph into two groups: those that are directly associated with metabolic reactions either as reactants, products, or enzymes; and all others. Each group is then further divided into its connected components, and each component is laid out individually in a third graphG3 (the Explanation Graph), the only graph that is visible to the user. For each metabolic connected component, we assemble the set of associated reactions into a temporary pathway, and then lay out the pathway using the automated metabolic pathway layout algorithms that are an integral part of Pathway Tools software. The non-metabolic components will have a one-to-one correspondence between the nodes in *G*2 and the nodes in the diagram, so each of these sets are copied to *G*3 and laid out as a tree (with some strategic temporary hiding or reversing of edges while running the layout algorithm to make the topology of a graph that is not a tree more tree-like). Finally, we lay out all components relative to each other to produce the final diagram. We use different arrowhead styles to indicate different types of influence, and, for non-metabolic interactions, edge colors indicate whether an influence is activating or inhibiting. We also use color to highlight all entities in the supplied entity sets (including the covering set entities, if they are not part of the original target set).

Figure 2d shows the Explanation Graph *G*3 generated from the example filtered graph *G*2 in Fig. 2c. This graph was constructed hierarchically from three components: (1) the metabolic pathway consisting of reactions R1, R2 and R3; (2) the metabolic pathway consisting of reaction R5; and (3) the connected transcription factors TF0 and TF1. Note that even though M1 and M3 are not present in *G*2, they must be included in *G*3 to show the reactions catalyzed by E1 and E3.

The user can mouse over any node or edge in the figure to see more information. Clicking on a node or edge causes the relevant entity to be displayed in the main Pathway Tools display window.

### Additional GUI operations

In the graphical user interface for the MultiOmics Explainer, a control panel to the left of the pane containing the Explanation Graph provides additional information and options. It lists any entities in the target sets for which no connections to any other entities were found. It also provides a checklist of entities for which connections were found. Users can select a subset of the original set and regenerate the display to only show connections between the selected entities. This feature is particularly useful in cases where the size and interconnectedness of the filtered graph makes it impossible to generate a display that makes it easy to visually follow all the connections (in some cases, this will be due to the limitations of our automated pathway layout algorithms in handling highly interconnected reaction networks; in other cases, this will be due simply to the visual clutter caused by large numbers of edges). Reducing the number of selected entities can result in a clearer diagram. When the display is regenerated with a new entity subset, *G*2 and *G*3 are recreated from scratch, but *G*1 is unchanged.

In directed mode, if parity information (increase or decrease in abundance or activity) was supplied for both conditions and effects, then we can determine which, if any, of the paths connecting a condition/effect pair are consistent with their respective parities. When one or more consistent paths exist in *G*2 for a pair, that pair is listed in the control panel. A button will bring up a textual description of such paths to complement the diagram. An example is shown in Fig. 3. It should be noted that when a graph involves one or more reversible reactions, paths can often be generated to support either parity.

In undirected mode, the list of all influencers in the top-scoring covering set are listed, as well as the top ten influencers in general. These influencers are presented as a selectable list, as shown in Fig. 6, so you can choose to regenerate the figure to show connections to influencers that are not part of the default set. In addition, you can bring up a menu of all other influencers sorted by score, if you wish to see connections to any influencer that is not in the top ten.

## Results

To test the utility of the MultiOmics Explainer, we searched the *E. coli* literature for example omics datasets that produced a relatively small number of interesting genes and/or metabolites in need of an explanation.

### Directed mode examples

Fuhrer et al. [1] have conducted a systematic genome-wide examination of the response of more than 7000 metabolite concentrations to more than 3800 *E. coli* K–12 single-gene deletions. For every metabolite, they were therefore able to identify a set of genes whose deletion significantly impacts that metabolite. We took some of the examples presented in their paper and attempted to explain them using the MultiOmics Explainer. In these cases, the deleted genes are the conditions, and the metabolite is the effect. Note that their paper speaks of these examples in the most general terms only, so does not propose specific paths that we could compare our results to.

**Fig. 4:**
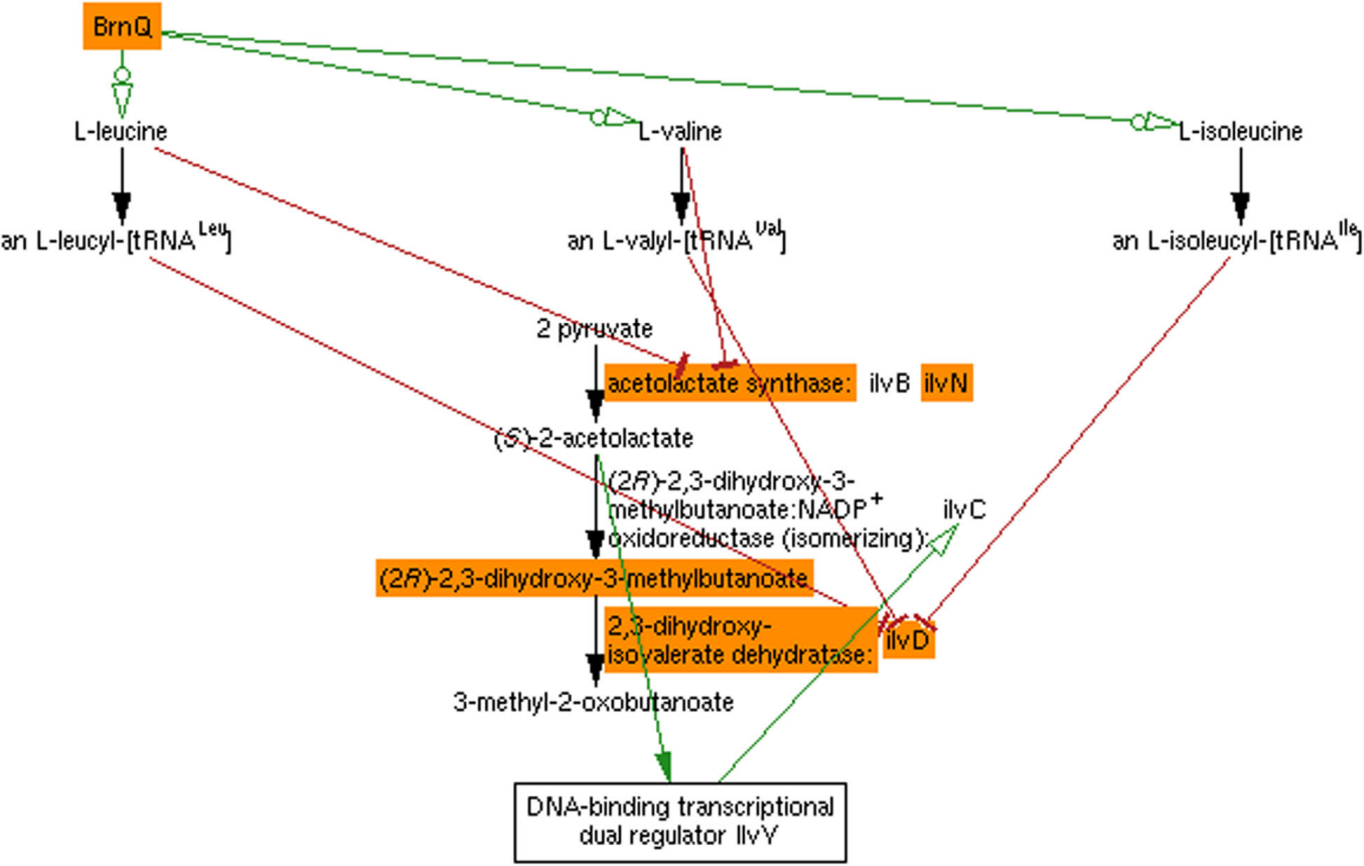
A diagram generated by the MultiOmics Explainer showing the paths of influence to dihydroxy-methylbutanoate from the genes found to significantly impact it. The altered genes, their enzyme products, and dihydroxy-methylbutanoate are all highlighted in orange. Green arrows represent activation or promotion, blunt red arrows represent inhibition, and black arrows represent chemical reactions. The different arrowheads on the green arrows indicate different modes of action. The arrowheads with circles originating from BrnQ indicate that BrnQ transports (or otherwise catalyzes an increase of) the three listed amino acids. The filled arrowhead from 2-acetolactate to transcriptional regulator IlvY indicates that 2-acetolactate is a component of the active regulator complex. The unfilled arrowhead from IlvY to the ilvC gene indicates transcriptional or translational activation of the gene, which codes for the enzyme to its left

The authors found that most significant changes can be explained by highly local paths within the network, with knocked-out genes most commonly affecting metabolites fewer than three steps away in the metabolic network. It is no surprise that the MultOmics Explainer is able to recapitulate these relationships. However, even at a relatively close metabolic distance, the inclusion of regulatory relationships enables us to detect paths of influence that would typically have required human inspection and inference. An example is the case of dihydroxy-methylbutanoate, shown in Fig. 4. In addition to the direct impact on dihydroxy-methylbutanoate levels by IlvD and IlvN, at metabolic distances of 0 and 1, respectively, the diagram generated by the MultiOmics Explainer shows how the transporter BrnQ impacts dihydroxy-methylbutanoate, in that the amino acids it imports directly inhibit the enzymes that produce acetolactate, and attenuate expression of IlvD. The diagram also shows how acetolactate promotes its own conversion to dihydroxy-methylbutanoate by binding to the transcription factor IlvY to activate expression of the enzyme for the conversion, IlvC. These inferences would not have been possible without a database that encodes each of these different types of interactions.

However, the authors also point out cases where the metabolic distance between the genes and affected metabolites is quite large. In one well-understood case, the authors make mention of how several genes related to iron accumulation affect the production of *cis*-aconitate, by virtue of an iron-sulfur cluster being a required cofactor of the aconitase enzymes AcnA and AcnB. Figure 3 shows how our tool correctly identifies and illustrates this same relationship. It is also interesting to note what was not found by our tool. No relationship was found between EntB, an enzyme involved in enterobactin biosynthesis, and *cis*-aconitate, which seems counterintuitive given that the ferric enterobactin complex is clearly present in the graph. Further inspection revealed that currently EcoCyc contains no reaction that can convert enterobactin to ferric enterobactin, since this is a spontaneous reaction that occurs outside of the cell. Thus, this creates an inadvertent gap in the metabolic network. The same explanation likely applies to the failure to find a link to AroB, since chorismate is the precursor to the enterobactin biosynthesis pathway.

Another case of distal effects cited by the authors is the impact of the aro and pur genes on malate levels. The authors state that there are no known regulatory relationships that can explain these relationships, and their cause is not well understood. Our tool suggests paths through the metabolic network by which these genes are connected to malate (Additional file 1: Figure S1), but it is not clear how much biological significance the suggested paths might have.

Khodursky et al. [15] conducted a series of gene expression experiments to identify *E. coli* genes with significantly changed expression profiles under conditions of excess tryptophan, tryptophan starvation, and/or disabling of the trpR repressor gene. To evaluate the quality of the results generated by our tool, we ran the MultiOmics Explainer on a total of 120 genes identified by these experiments. In these cases, the condition was either tryptophan or trpR, and the effects were the set of significantly changed genes. For each of the 81 genes in which a possible route between condition and effect was identified, we asked a biologist well-versed in *E. coli* genes and metabolism to estimate the likely biological significance of the suggested routes.

**Fig. 5:**
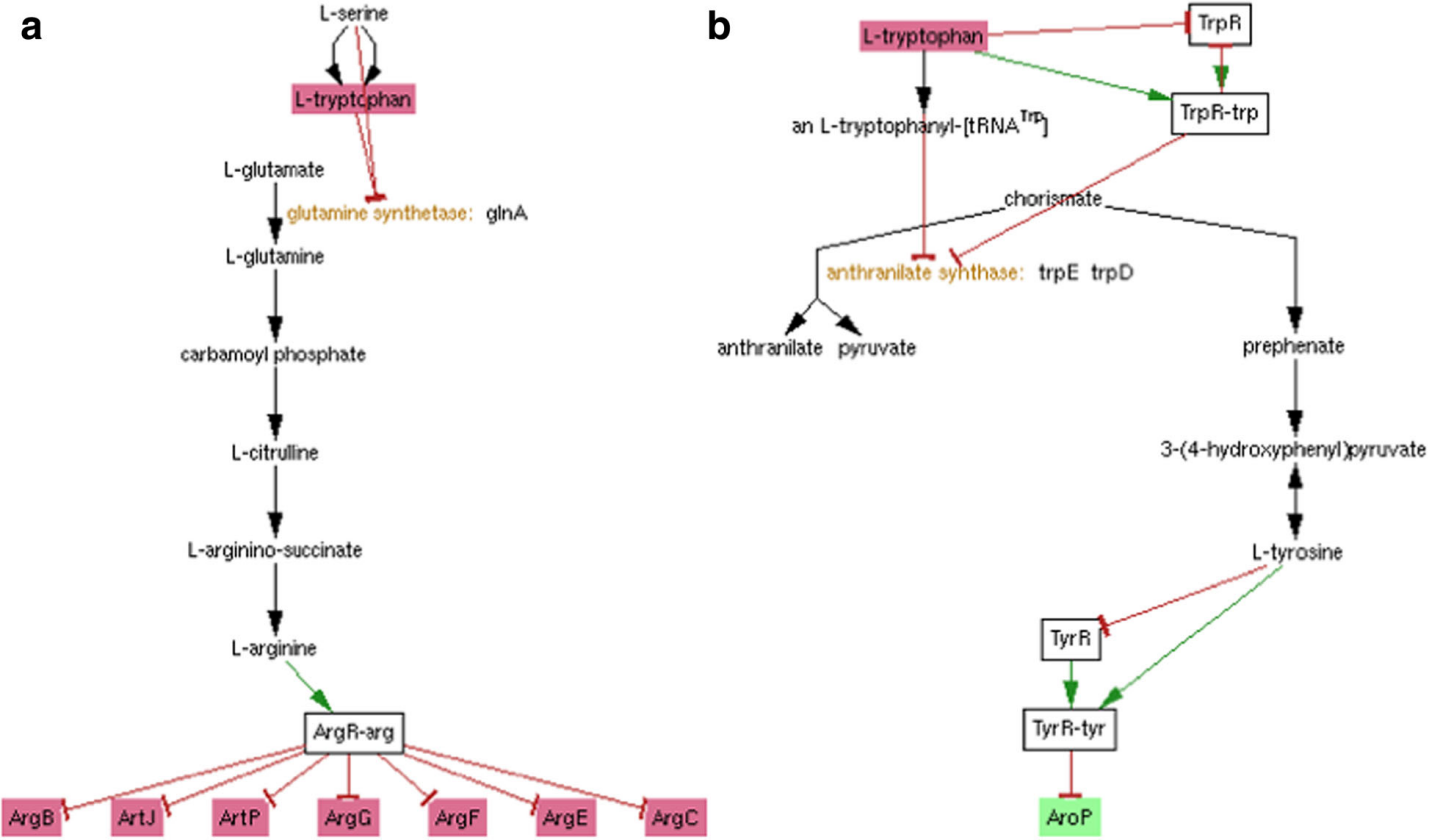
Examples of two sets of routes generated by the MultiOmics Explainer that were considered to be both non-obvious and of likely biological relevance. a) Tryptophan inhibits the enzyme glutamine synthetase, which affects the production of arginine and therefore the expression of the ArgR regulon. b) Tryptophan inhibits enzyme anthranilate synthase (directly, by attenuation, and by TrpR repression), affecting the extent to which chorismate is directed towards production of tyrosine, which inhibits expression of AroP

For 25 genes, the suggested routes were deemed of likely or probable biological relevance. Ten of these (those genes directly regulated by tryptophan or TrpR) were considered “obvious”, but 15 of them were not obvious and were therefore potentially interesting. Two routes, covering several of these genes, are shown in Fig. 5. In particular, Khodursky *et al* claim that prior to this study, the impact of tryptophan starvation on the arginine biosynthetic genes was not anticipated, so the suggestion of a mechanism by which this occurs would have been valuable. For 34 genes, the routes found by the algorithm were considered unlikely to be biologically relevant. Most of these were routes that passed through ppGpp, a global regulator that impacts expression of a large number of genes under conditions of environmental stress, but the direction of change of different genes was not consistent with primary regulation via ppGpp. For 22 genes, the biologist was not able to make a determination of the likely biological significance of the suggested routes—these routes were definitely not obvious and some of them might suggest hypotheses for further investigation. These results are summarized in Table 2 and in more detail in Additional file 1: Table S2.

**Fig. 6:**
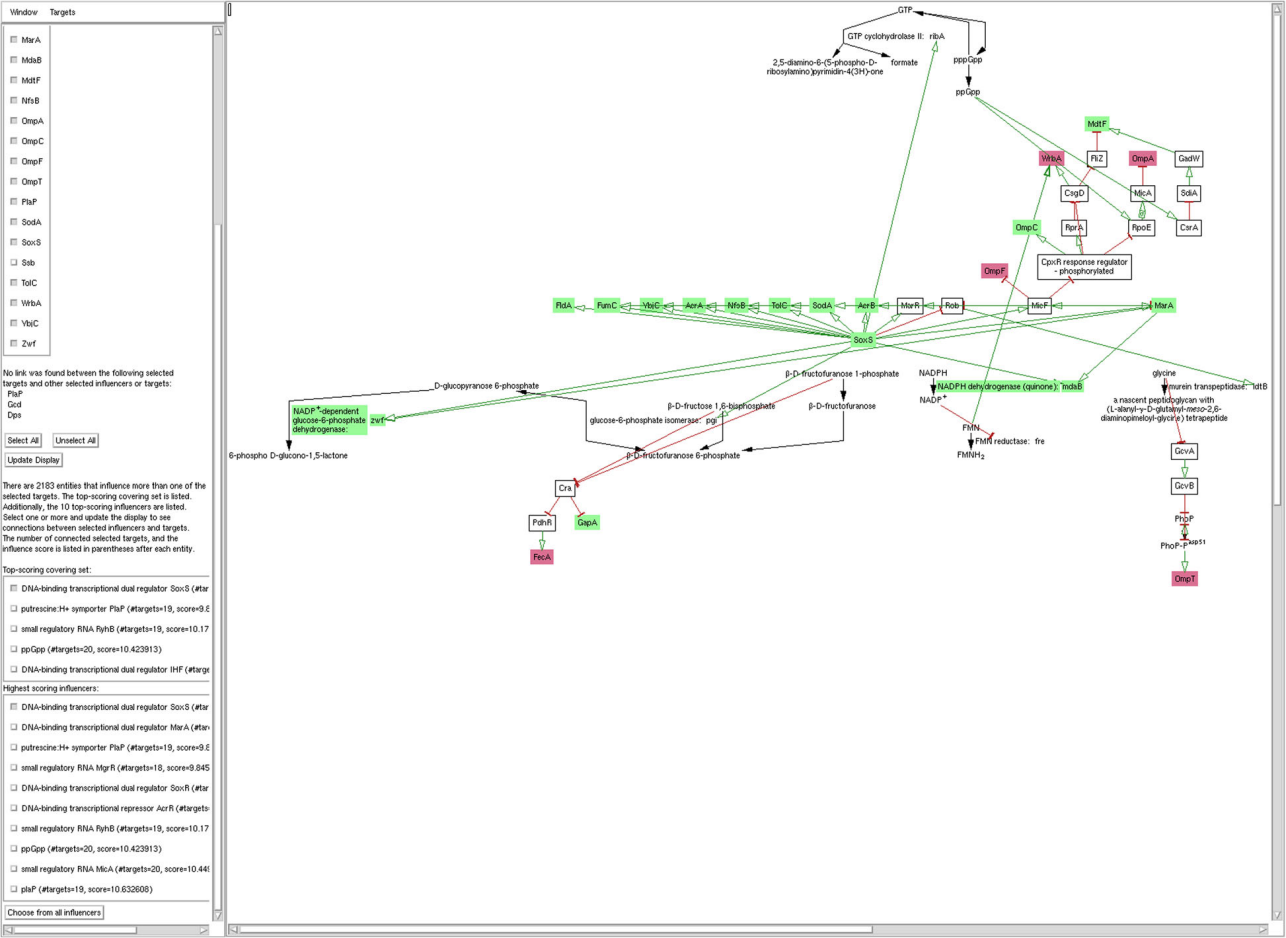
The MultiOmics Explainer operating in undirected mode on the benzylalkonium chloride dataset. Connections to the highest-scoring influencer, SoxS, only are shown (and one target gene, Ssb, is excluded, in order to reduce the figure to a reasonable size). Entities in green are target entities that show increased expression; entities in red show decreased expression. Green arrows represent activation, red arrows represent inhibition, and black arrows represent metabolic reactions

**Table 2:** Summary of an evaluation of the routes predicted by the MultiOmics Explainer connecting tryptophan to 120 genes with significantly changed expression levels

Result of evaluation	Number of genes
No route found	39
Route was obvious	10
Route was non-obvious and reasonable	15
Plausibility of route could not be determined	22
Route was unlikely to be biologically significant	34
Total	120

Traditional enrichment analysis (using the enrichment analysis tool available at BioCyc.org) indicates that this gene set is over-represented in amino acid biosynthesis genes, particularly for synthesis of arginine and the aromatic amino acids, and, to a lesser extent, cell locomotion genes, but is unable to suggest specific mechanistic links to tryptophan. Any of the pathway-based refinements of traditional methods depend upon the existence of defined signaling pathways involving these genes, which do not exist in EcoCyc. Our tool was highly effective at explaining the link from tryptophan to the amino acid biosynthesis genes, without depending on defined pathways (though of course there is overlap between some of the suggested routes and known metabolic pathways), and the suggested routes typically include both regulatory and metabolic steps, something no other tool can do. Our tool was less successful at identifying plausible routes for the locomotion-related genes. This evaluation highlights the challenge of distinguishing between biologically relevant and non-relevant routes, but also shows the value of the tool both for identifying known routes and for hypothesis generation.

### Undirected mode example

In Bore et al. [16], *E. coli* K–12 was incubated with successively higher doses of benzylalkonium chloride (BC), a commonly used disinfectant that kills bacterial cells, to produce three adapted strains. Transcriptional and proteomic analyses were then conducted on the adapted strains, to produce a list of 25 genes/proteins that were significantly differentially expressed in the adapted strains relative to the control strain. The purpose of the experiment was to shed light on the mechanisms by which BC and related compounds disrupt the cell and by which resistance is acquired.

Because the mechanistic action of BC is unknown, no mechanistic link exists between BC and any other entity in EcoCyc (in fact, BC is not present in EcoCyc at all). Thus, this study offers a useful example for testing out the MultiOmics Explainer in undirected mode. In this case, the input effects set is the set of 25 genes and proteins identified by the analysis, and the goal is to identify one or a small number of genes that can explain the observed set.

Figure 6 shows a result from the MultiOmics Explainer run on this dataset. Connections were found between all but one (RpsF) target genes. The top-scoring influencer, SoxS, was also among the target set (in fact, the authors identify it as the most highly upregulated gene in their experiments), and can be linked to 20 of the remaining 24 entities. This matches the conclusion drawn by the study authors that BC likely results in oxidative stress that can be ameliorated by the overproduction of SoxS. Figure 6 shows how SoxS is connected to most of the other genes in the target set. The authors also mention MarA as a lesser possible contributor. MarA was the second highest scoring influencer identified by our tool (MarA is regulated by SoxS, so everything connected to MarA is also connected to SoxS). Traditional enrichment analysis also identifies the SoxS and MarA regulons as significantly over-represented, but misses the connection to genes such as wrbA and mdtF, where there are multiple regulatory intermediates. The few remaining genes that are not connected to SoxS could represent alternate paths to BC resistance, or they could be the result of gaps in the EcoCyc regulatory network.

## Conclusions

The MultiOmics Explainer is a valuable tool to help researchers understand and interpret the results of their omics experiments in the context of what is known about an organisms’s metabolic and regulatory network. In directed mode, the user supplies two sets of entities, those that are changed as conditions of an experiment, and those that change as effects of the experiment, and the tool searches the network to identify paths by which the conditions influence the effects. This mode is most appropriate for experiments whose conditions can be reduced to changes to one or a few genes or metabolites, such as a gene-knockout experiment or a nutrient-substitution experiment. Undirected mode is best suited to experiments that either include conditions that do not map to specific entities or involve entities whose function is unknown (and thus are not part of the existing network). In this case, users specify the set of effect entities, and the tool identifies a set of potential influencers that can explain as much of the input set as possible. In both modes, the tool produces a diagram that summarizes the paths of influence and provides links back to the PGDB for more information.

It must be noted that operation of the MultiOmics Explainer depends upon the rich and extensive set of metabolic and regulatory relationships encoded within the EcoCyc database. Running our software on examples of omics experiments taken from the *E. coli* literature demonstrates that paths of influence often involve multiple types of interactions, combining metabolism; transport; enzyme cofactors and substrate-level regulators; transcriptional and translational regulation; and protein-ligand interactions. The MultiOmics Explainer highlights the value of EcoCyc, not just as a reference but as a basis for computational inference.

## Availability and requirements

The MultiOmics Explainer is a component of the Pathway Tools desktop application.

**Project name:** Pathway Tools

**Project home page:** http://bioinformatics.ai.sri.com/ptools/

**Operating systems:** Macintosh, Windows, and Linux

**Programming language:** Common Lisp

**Other requirements:** Pathway Tools version 22.5 or later

**License:** Freely available to academic and government researchers with signed license agreement. See https://BioCyc.org/download.shtml. Source code available upon request.

**Any restrictions to use by non-academics:** A license fee applies to commercial use. See https://BioCyc.org/download.shtml.

## Additional file

Additional file 1 Supplementary Material. (PDF 79 kb).

## Data Availability

The MultiOmics Explainer is a component of the Pathway Tools desktop application. The EcoCyc database is bundled with the Pathway Tools software. Databases for other organisms may require a paid subscription. To obtain the software, see https://BioCyc.org/download.shtml. The data used to generate the example displays were drawn from the published figures and tables in the cited papers.

## Abbreviations

BC: Benzylalkonium chloride
PGDB: Pathway/genome database
