# Supplementary material for: The MultiOmics Explainer: explaining omics results in the context of a pathway/genome database
Source: BMC Bioinformatics. 2019 Jul 18;20:399. doi: 10.1186/s12859-019-2971-6 (PMC6637615; doi:10.1186/s12859-019-2971-6)
Supplement: Supplementary file 1 — Supplementary Material. (PDF 79 kb). [file 12859_2019_2971_MOESM1_ESM.pdf]

Supplemental Table S1

Organism	RGs	TFs	REs	CEs
Escherichia coli K-12 substr. MG1655 (EcoCyc)	2050	319	631	588
Bacillus subtilis subtilis 168	1148	170	22	32
Escherichia coli O157:H7 str. EDL933	619	43	0	2
Shigella flexneri 2a str. 2457T	521	43	0	0
Caulobacter crescentus NA1000	400	4	0	0
Vibrio cholerae O1 biovar El Tor str. N16961	390	17	0	5
Listeria monocytogenes 10403S	241	14	0	0
Yersinia pseudotuberculosis IP 32953	239	20	0	1
Escherichia coli K-12 substr. RV308	175	25	0	0
Streptomyces coelicolor A3(2)	107	32	1	1

Supplemental Table S1: The organisms within BioCyc that have the greatest amount of regulatory data encoded. RGs (regulated genes): the number of genes for which the DB specifies at least one transcriptional or translational regulator. TFs: the number of transcription factors or other transcriptional or translational regulators. REs (regulated enzymes): the number of enzymes with substrate-level activators or inhibitors. CEs (cofactor enzymes): the number of enzymes with specified cofactors.

Supplemental Table S2

Gene	Result	Notes on identified routes
ampE, ettA, evgA, fecB, fecC, fecD, gpr, lacA, murJ, pheA, plaP, rbsC, recD, rffH, ridA, rpnE, sieB, stfR, tciF, tciG, tfaR, yafD, yafE, yaiT, yciF, yciG, tciT, ycjG, ycjX, ycjY, ydhR, yedF, yhdJ, yhjE, yjiT, yjjX, ymgG,yqhA, ytjC	not found	
aroH, mtr, tnaA, tnaB, trpA, trpB, trpC, trpD, trpE, trpR	obvious	
argA, argB, argC, argD, argE, argF, argG, artJ, artP	reasonable	via glnA, arg, ArgR
tyrR		via tnaA, tyrosine
leuB		via tyrosine
fimC, fimD, nadE		via tyrosine, Lrp
aroP		via tyrosine, TyrR
nupC	unknown	via cytidine
pinQ, pinR, tfaQ		via enterobactin, Fur
mgtA, ompT, ydeO		via glycine, GcvA
cysJ, hybA, hybB, modA		via O-acetyl-L-serine, CysB
narP		via O-acetyl-L-serine, CysB, NarX
aroF, tyrA		via PRPP, hypoxanthine
carA, codA, codB, glnG, hisG		via PRPP, hypoxanthine, PurR
patA		via pyridoxal 5′-phosphate
malE		via pyridoxal 5′-phosphate, malP
appY		via serA, gltA, DpiB
gntP	unlikely	via D-fructuronate, UxuR
argI, asnB, csgD, fabA, flgB, flgI, flgJ, gltJ, gltK, katE, oppC, osmY, otsB, poxB, rplA, rplK, rpsU, setA, talA, tufB, wrbA, ybeL, ydeN, ydiV, yeaG		via GMP, ppGpp
cheB, cheZ, fliA, fliI, fliJ, fliS, rpmA		via GMP, ppGpp or kbl, GcvA
xylF		via GMP, ppGpp, RpoE

Supplemental Table S2: The results of a biologist's evaluation of the routes predicted by the Multi-Omics Explainer connecting tryptophan or trpR to 120 genes with significantly changed expression levels. The notes in the 3rd column are not complete descriptions of the predicted routes, but rather an indicator of key intermediates used to group the genes into sets that were evaluated together.

Supplemental Figure S1

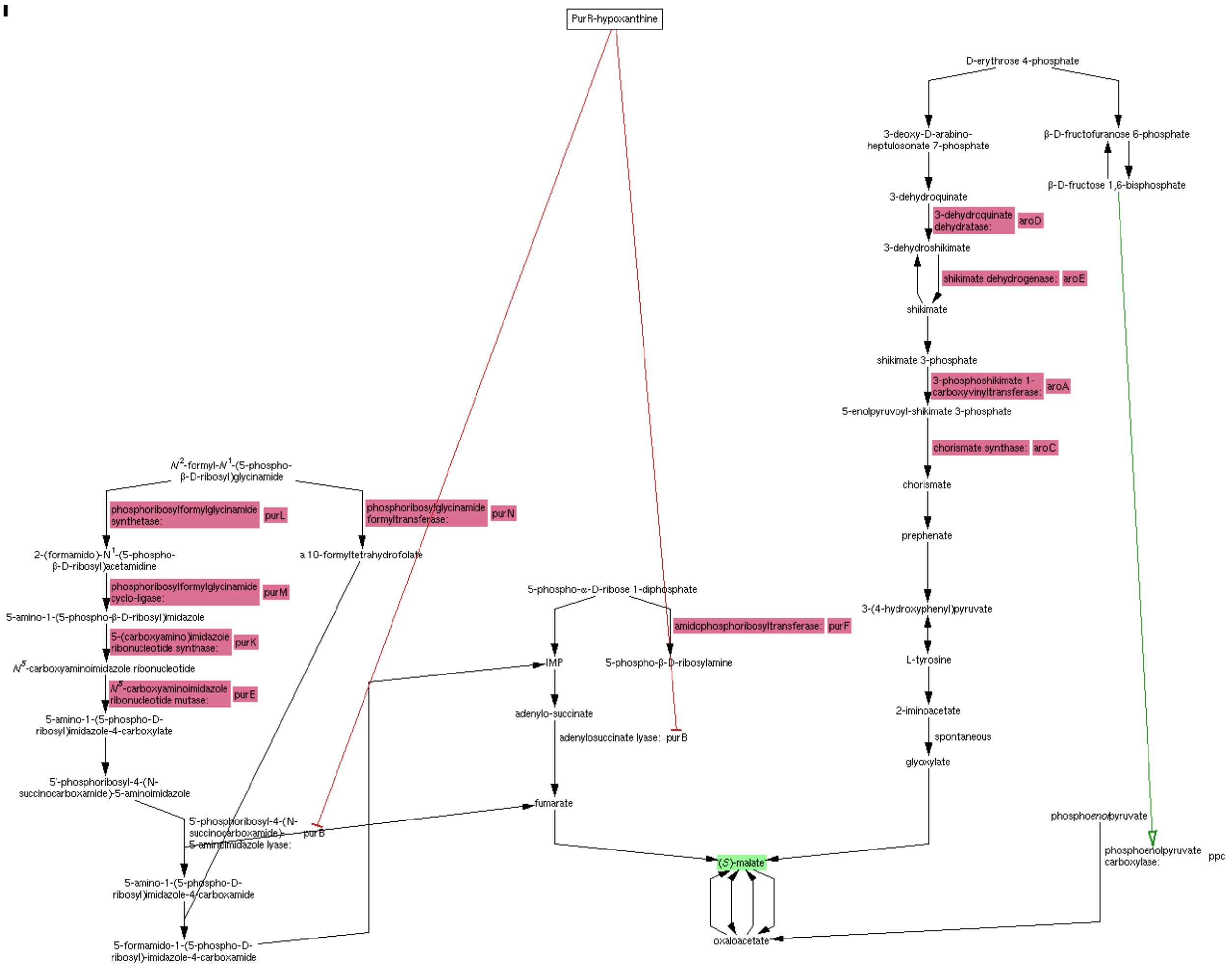

Supplemental Figure S1: A diagram generated by the MultiOmics Explainer suggesting paths by which the highlighted aro and pur genes might influence malate, albeit of dubious biological significance.
